# Perspectives on Influenza Vaccination Among COVID-19 Unvaccinated Patients in US Emergency Departments: A Qualitative Study

**DOI:** 10.5811/westjem.62236

**Published:** 2026-10-29

**Authors:** Cindy Li, Jaran White, Anna Tupetz, Alena Pauley, Rachel E. Geyer, Kelli N. O’Laughlin, Herbert C. Duber, Kristin Rising, Alexzandra T. Gentsch, Robert M. Rodriguez, Stephanie A. Eucker

**Affiliations:** * University of Colorado, Anschutz School of Medicine, Aurora, Colorado; † University of North Carolina at Chapel Hill, Department of Psychology and Neuroscience, Chapel Hill, North Carolina; ‡ Duke University, Department of Emergency Medicine, Durham, North Carolina; § University of California San Diego, University of California San Diego School of Medicine, La Jolla, California; ¶ University of Washington, Department of Emergency Medicine, Seattle, Washington; || University of Washington, Department of Family Medicine, Seattle, Washington; # University of Washington, Department of Global Health, Seattle, Washington; ** Institute for Health Metrics and Evaluation, Department of Health Metrics Science, Seattle, Washington; †† Thomas Jefferson University, Sidney Kimmel Medical College, Department of Emergency Medicine, Philadelphia, Pennsylvania; ‡‡ Thomas Jefferson University, Center for Connected Care, Philadelphia, Pennsylvania; §§ University of California Riverside, Department of Emergency Medicine, Riverside, California; |||| Durham Veterans Affairs Medical Center, Emergency Department, Durham, North Carolina

## Abstract

**Introduction:**

Efforts to increase influenza vaccine acceptance must consider the importance of patient education and familiarity with vaccinations and influenza in the emergency department (ED), particularly for populations without another primary source of healthcare. This is the first study, to our knowledge, to assess in-depth perceptions regarding the influenza vaccine and identify barriers and facilitators to influenza vaccination among COVID-19 unvaccinated adult ED patients.

**Methods:**

We conducted a qualitative idiographic interview study from July 12, 2021, to September 24, 2021, at four EDs in urban cities across the United States. We recruited adult ED patients (18 years and older) unvaccinated for COVID-19, without a primary care clinician, who were fluent in either English or Spanish. The primary finding was a detailed understanding of patient perceptions regarding the influenza vaccine, with secondary findings assessing influenza vaccination rates, barriers and facilitators to being vaccinated, sources of information, and future vaccination intentions. We used a content analysis approach to identify themes around patient experiences, concerns, barriers, information sources, and future intentions regarding influenza vaccination.

**Results:**

Of 65 participants interviewed, 48 discussed the influenza vaccine and were included in this analysis. Among these participants, the median age was 34 years, 21 (43.8%) were women, and 14 (29.2%) were Hispanic. We found three core themes influencing influenza vaccine hesitancy: 1) previous experiences with influenza illness and/or the vaccine; 2) knowledge about the influenza vaccine; and 3) confidence in vaccine safety and effectiveness. In our cohort, 10 (20.8%) participants demonstrated knowledge of the purpose and mechanism of the influenza vaccine. In addition, 14 (29.2%) patients suggested they would accept the vaccine if offered, while 18 (37.5%) patients stated they did not plan to receive the influenza vaccine and/or expressed strong hesitancy due to concerns about lack of vaccine effectiveness, fear of side effects, and lack of concern regarding influenza illness.

**Conclusion:**

Our findings identify an important barrier that could be overcome by ED-based vaccine education, particularly for underserved patient populations. Interventions to increase influenza vaccine uptake could focus on overcoming hesitancy related to prior experiences and understanding of vaccine safety and effectiveness.

## INTRODUCTION

Vaccine hesitancy is defined by the Sage Working Group on Vaccine Hesitancy as “a delay in acceptance or refusal of vaccination despite the availability of vaccination services.”^1^ Vaccine hesitancy, as a contributor to influenza illness, was cited as one of the top 10 threats to global health by the World Health Organization as recently as 2019. During the 2023–2024 season, influenza accounted for 18 million healthcare visits, 470,000 hospitalizations, and 28,000 deaths.^2^ During influenza seasons, emergency departments (ED) consistently experience higher patient census, with longer wait times, more admission holds, and poorer patient outcomes generally linked to an increase in flu-related ED visits.

In November 2022, the American College of Emergency Physicians, along with other organizations, authored a letter to the U.S. President Joseph Biden describing the threat that ED admission holds—known as “boarding”—place on the healthcare system, painting “a picture of an emergency care system already near collapse.” Specifically, this letter highlighted a “triple-threat” that influenza, respiratory syncytial virus (RSV), and COVID-19 pose, in addition to the lack of inpatient hospital beds overwhelming EDs.^3^–^5^ In the same month, the American Academy of Pediatrics and the Children’s Hospital Association urged the president to declare a national emergency due to rising influenza rates and an unprecedented surge in RSV among the pediatric population, with multiple states reporting hospitalizations exceeding 100% of their pediatric bed capacity.^6^,^7^ Thus, there is an urgent need to prevent influenza-related hospitalizations through public health efforts.

Influenza vaccines have been identified as the most effective method of mitigating rates of symptomatic influenza illness, hospitalization, and mortality.^8^ Furthermore, influenza vaccination has been associated with lower odds of severe COVID-19 outcomes such as hospitalization and death.^8^–^10^ Despite this evidence, in the 2024–2025 season only 44.0% of U.S. adults and 44.5% of children were vaccinated against influenza.^11^ Additionally, a 2025 study demonstrated that influenza vaccination rates decreased by 6.41 percentage points post-pandemic relative to pre–COVID-19 levels.^12^ Recent U.S. statistics show that influenza vaccine history is closely linked to COVID-19 vaccine acceptance, with influenza vaccinators significantly more likely to receive COVID-19 vaccination.^13^,^14^

Issues with low vaccination rates and vaccine hesitancy are further complicated by racial, ethnic, and socioeconomic disparities in the U.S. Non-Hispanic White and Asian individuals have higher vaccination rates than those of other populations, and Hispanic and Black individuals demonstrate the lowest rates among the public.^15^–^17^ Low-income status relative to the poverty level has been identified as one of the strongest factors associated with influenza vaccine hesitancy and poor influenza outcomes.^18^ Compounding this, individuals from low socioeconomic backgrounds experience a greater burden related to illnesses like influenza due to rapid spread of the virus in close living quarters, limited access to healthcare services, and employment that penalizes workers for missed workdays.^18^ Additional factors that contribute to influenza and COVID-19 vaccine hesitancy include lower level of education, younger age, and lack of healthcare insurance.^19^–^23^ Thus, efforts to increase influenza vaccination rates must address the differential impacts of vaccine hesitancy and influenza illness stemming from social determinants of health and increase vaccine acceptance among these underserved populations.

**Population Health Research Capsule**

What do we already know about this issue?

*Influenza vaccine hesitancy and poor influenza outcomes correlate with socioeconomic factors common among emergency department (ED) patient populations.*

What was the research question?

*We sought to qualitatively characterize perceptions of, barriers to, and facilitators of influenza vaccination among COVID-19 unvaccinated adult ED patients.*

What was the major finding of the study?

*Barriers to influenza vaccination among ED patients included concerns about vaccine effectiveness, side effects, and lack of concern regarding influenza illness.*

How does this improve population health?

*Our detailed findings offer valuable insights to inform ED-based education interventions to improve influenza vaccine uptake among vulnerable populations.*

The ED serves all patients as a safety net within the broader U.S. healthcare system, with patients from lower-income backgrounds, lower educational attainment, and without healthcare coverage disproportionately receiving care in the ED.^24^,^25^ The ED population also comprises patients with no primary care clinician, who thus have little to no access to the healthcare system beyond the ED. As such, the ED may serve as a critical site for delivering both information and influenza vaccination to populations that are typically the most vaccine-hesitant and under-vaccinated, thereby effectively addressing disparities and providing equitable access to influenza vaccination and vaccine education.

Existing studies aiming to understand influenza vaccine hesitancy in adult ED populations have primarily used one of two methodologies: quantitative designs to measure vaccine uptake; or interventional studies to increase vaccine willingness, which may involve preintervention and postintervention feedback.^26^–^29^ These studies have consistently demonstrated that hesitancy is a major barrier to vaccination and that educational interventions in the ED can improve vaccination rates. However, to our knowledge, no studies have used in-depth qualitative analysis to understand the key beliefs, experiences, and concerns that underlie vaccine hesitancy among adult ED patients. The qualitative methodology is essential for identifying and characterizing, with greater nuance, the variety of factors driving hesitancy, and it is needed to inform the design of future interventions to improve vaccination efforts.

In response to COVID-19 and influenza vaccine disparities among diverse populations, including historically underserved communities, we developed a research program, Promotion of COVID-19 Vaccination in the Emergency Department (PROCOVAXED). We conducted in-depth qualitative interviews of vaccine-hesitant patients whose primary healthcare access occurs in EDs and then created population-specific COVID-19 vaccine messaging platforms (videos, informational flyers, and scripts for face-to-face emergency clinician messaging) to address identified COVID-19 vaccine concerns and promote vaccine uptake.^30^ Subsequently, in a cluster-randomized controlled trial, we demonstrated that implementation of COVID-19 vaccine messaging platforms in EDs led to greater COVID-19 vaccine acceptance and uptake among unvaccinated ED patients, with larger effect sizes among Hispanic participants and those without primary care.^31^ To address the similarly important topic of low influenza vaccine uptake in underserved ED populations, we analyzed influenza-related interviews conducted as part of our overall PROCOVAXED study to explore patient perceptions specific to influenza vaccine willingness and hesitancy.

## METHODS

In this descriptive idiographic qualitative study, we conducted semistructured interviews with ED patients who had not received a COVID-19 vaccine and were enrolled in a parent study, PROCOVAXED (clinicaltrials.gov ID: NCT05142332).^32^ The study was approved by institutional review boards (IRB) at each study site, with a central IRB overseeing study activity. The recruitment period for this study extended from July 12, 2021, to September 24, 2021. The study was conducted within four urban EDs at hospitals located in four U.S. states. Research personnel used electronic health records to screen patients who were potentially eligible and actively seeking treatment within these EDs. Inclusion criteria included adults (≥18 years of age) with no primary care visits in the prior year who had not yet received a COVID-19 vaccine. Exclusion criteria were as follows: being unable to consent (consent consisted of the patient being clinically sober and able to fully understand the risks and benefits of the study); living in a nursing facility; being incarcerated; experiencing trauma or psychiatric chief complaint; being under suspicion of acute COVID-19 illness; and being unable to communicate in English or Spanish. Prior knowledge about either the COVID-19 or influenza vaccines was not required for participation. Research personnel approached potential participants to screen for eligibility and interest, and each participant provided written informed consent.

Interview guides were developed using existing literature and data from our REVVED UP survey study,^23^ and were reviewed by both the study team and the COVID-19 Research Patient and Community Advisory Board located at the central institution.^23^,^32^ Following instruction and training from coinvestigators in interview techniques and data collection, research coordinators obtained participant consent and conducted qualitative interviews in the ED. Questions during the interview focused on perceptions, trusted messengers for vaccine communication, and barriers participants may face to the COVID-19 vaccination, and subsequently included a second section exploring participants’ experiences with and opinions of influenza and influenza vaccines, the data from which are the focus of this analysis. Spanish interviews were conducted by a certified Spanish-speaking team member.

The study team always attempted to ask all participants questions about the influenza vaccine; however, this was not always possible due to time constraints and the primary focus on COVID-19 vaccines. All participants who completed any portion of the influenza component of the interview were included in the present study. Interviews were performed using semistructured guides (Table 1) and recorded on handheld audio recorders. Each participant’s demographic information was entered into a secure Research Electronic Data Capture (REDCap) database. The sample size was not predetermined, and participant enrollment continued until thematic saturation of the primary interview guide was achieved, as evaluated through transcription assessment at regular intervals. There was no independent enrollment target for the influenza-focused portion of the study.

### Data Analysis

For this substudy, we conducted a content analysis of all data identified in the parent study as pertaining to influenza and influenza vaccination.^33^ We created the codebook (Table 2) to analyze this influenza-specific data using inductive and deductive coding. The initial codebook used the same themes and codes identified in the COVID-19 data from the parent study, given the similarity of questions, and additional emergent codes were added for topics unique to the influenza-related responses. Each code was explicitly defined, with detailed descriptions and examples. Two independent reviewers conducted the coding process—each reviewed all material and assigned quotes to codes, then identified any inconsistencies in coding, and met to discuss and resolve discrepancies.

## RESULTS

We interviewed 65 participants across all sites in the PROCOVAXED parent study, of whom 48 participants (73.8%) completed the portion of the interview relating to influenza and/or influenza vaccination and are included in these results. Of these, 27 (56.2%) identified as male, 14 (29.2%) identified as Hispanic, and 6 (14%) were interviewed in Spanish (Table 3). Themes, codes, and selected quotes from participant responses are available in Table 4.

### Theme 1: Experience with Past Influenza Illness and Vaccinations

Of the 48 participants included in this analysis, 3 individuals stated they had not heard of the influenza vaccine at the time of their interview. Eight participants received the vaccine annually, seven previously received the vaccine but no longer do, and 12 did not receive and had not ever received the vaccine.

When discussing reasons for either accepting or refusing the influenza vaccine, most participants described experiences (whether their own or of others) of contracting influenza and/or receiving vaccinations in years past. Six patients cited prior experiences contracting influenza after receiving the vaccine, “I noticed…every time I got vaccinated for the flu I ended up getting sick—end up getting the flu” (male, 34 years old [y/o)). Six other patients described experiences with adverse side effects after vaccination, thus leading to their hesitancy or refusal to continue receiving the vaccine, “I’ve gotten sick from [the flu vaccine], too. But I don’t take no more flu shots” (male, 42 y/o). Five individuals were unmotivated to receive the vaccine because they had never contracted influenza and did not think it was necessary, “…why would I get a vaccine for something I never caught” (male, 28 y/o).

### Theme 2: Knowledge and Concerns About the Vaccine

Ten participants reported a general understanding of the vaccine itself; for example, “…I’ve heard they’re injecting you with the virus to help get your body immune to it” (female, 28 y/o). Three individuals acknowledged that they did not know much about the vaccine other than, “Well, they just tell me to get it…every year they say go get your flu vaccine” (male, 59 y/o). Two people were unsure whether the vaccine makes the illness milder.

Many individuals also shared their concerns regarding influenza vaccine effectiveness and potential adverse side effects. Six participants did not believe that the vaccine was effective, saying “The flu vaccine’s been out for how many years?...older than I am, and it’s uneffective” (male, 28 y/o). Others thought that the vaccine could allow the virus to mutate: “…only next year that vaccine that they had put out antigens which made the flu stronger…it builds on itself type of a thing” (male, 52 y/o). Importantly, this idea also supported a belief that the COVID-19 vaccine would ultimately be ineffective. Four individuals reported greater trust in the influenza vaccine when compared to the COVID-19 vaccine, “at least it has a track history, it has something we can follow and see what effects it’s had on – through the years” (male, 51 y/o). Eight individuals mentioned their fear or worry about side effects such as fevers, other flu-like symptoms, and even longer-term disability, “I don’t know what kind of reaction I might have” (female, 37 y/o). Two patients expressed concern regarding the frequency of the vaccine, “I don’t want to be injected every year with stuff being poured into my body” (female, 30 y/o).” Others cited a fear of the “unknown” or a “foreign” substance, eg, “I’m not gonna put something foreign in my body that I don’t know where it came from, you know?” (male, 51 y/o).

### Theme 3: Sources of Information

Participants most commonly mentioned friends and family (word-of-mouth) and their personal experiences as reliable sources of information about the vaccine. Two individuals mentioned their experiences working in a healthcare setting, eg, “Because I’m an old x-ray technologist, I’ve always been cautious. I know how disease and viruses spread” (male, 67 y/o). One individual reported knowing a healthcare professional who personally refused to receive the vaccine, citing this as an influence in their decision-making. Many also mentioned hearing about the vaccine (including concerning stories of adverse side effects) in the news and social media but did not specifically identify these as trusted sources.

### Theme 4: Vaccine Access and Barriers

Most participants reported that the influenza vaccine was accessible to them and that they knew where to receive one if they wanted it. Although 27 patients had been previously offered the vaccine, 2 had not. Of those who were prompted to discuss specific barriers to accessing the vaccine, only one participant identified a barrier, which was lack of insurance. When asked whether there were obstacles to getting the vaccine, one subject stated, “Not at all. I get offered all the time” (male, 42 y/o).

### Theme 5: Future Intentions and Decisions

Despite widespread reported understanding of and access to the influenza vaccine, 19 participants stated that they did not plan to receive the vaccine in the future, 5 were strongly hesitant and/or likely to decline the vaccine, and 2 did not know enough about the vaccine to state their future intention. Many cited the same reasons they had for not vaccinating in the past including concerns about side effects, disbelief in the vaccine’s effectiveness, not trusting the contents of the vaccine, and rarely getting sick. For example, “I just haven’t had the flu ever since—when I stopped getting the flu vaccine, I stopped getting the flu” (male, 34 y/o). One individual stated their reason not to vaccinate was their pregnancy.

Conversely, 14 individuals clearly stated that they would accept the influenza vaccine. One additional participant stated that they were previously hesitant, but now would be more likely to accept the vaccine, stating, “I refused it at first, but then again in my profession, you’re around so many people” (male, 48 y/o).

The remaining responses did not contain sufficient information to identify their future intentions on whether to receive the influenza vaccine.

## DISCUSSION

Our study addresses a critical gap in knowledge regarding influenza vaccine hesitancy among the diverse patient populations served by the ED. The overarching reasons for vaccine hesitancy among our participants were a belief that the vaccine is not effective, fear of vaccine side effects, and a general lack of concern regarding influenza illness. Similar reasons have also been shown in previous literature for both influenza and COVID-19 vaccine hesitancy in ED and non-ED populations.^21^,^34^–^36^ Our participants cited prior experiences of suffering from influenza-like illness following vaccination as a major influence in their decision-making, with many patients directly attributing the vaccine to causing influenza, a commonly cited belief in other studies.^23^,^37^ Data from the Centers for Disease Control and Prevention 2026 RespVaxView report were consistent with our findings, suggesting a general lack of concern regarding influenza illness as a predominant driver in adults remaining unvaccinated, whereas the most common motivator towards vaccination was “to protect myself.”^38^ Similarly, adults expressing moderate to high concern over influenza were much more likely to vaccinate, compared to adults expressing little concern.^39^ Our findings also aligned with quantitative studies examining other populations, such as parental decision-making regarding vaccination of their child both before and after the COVID-19 pandemic, which similarly identified a lack of concern regarding influenza illness, concerns regarding vaccine safety/side effects, and doubts regarding vaccine effectiveness as primary contributors to vaccine hesitancy.^40^

Our results revealed overlapping themes with the parent study, which focuses on perspectives regarding COVID-19 vaccination.^32^ In the parent study, the majority of participants similarly expressed an overall lack of knowledge about the COVID-19 vaccine, as well as fear of long-term side effects and future health complications associated with vaccination. However, our findings regarding influenza vaccination differed in several major ways: 1) participants cited substantially fewer barriers to access relative to the COVID-19 vaccine; 2) participants highlighted word-of-mouth and personal experience as their most trusted sources of information regarding influenza vaccination, rather than doctors which were the most trusted source for the COVID-19 vaccine; and 3) distrust in government and pharmaceutical companies was a more prominent theme for COVID-19 than for influenza vaccination. These similarities and differences highlight how beliefs about influenza and COVID-19 vaccines may be inter-related and mutually driven, with the overall degree of hesitancy exacerbated by economic, cultural, and sociopolitical conditions related to the COVID-19 pandemic. In fact, recent data has emerged demonstrating that receipt of a COVID-19 vaccine is strongly associated with influenza vaccination with an adjusted odds ratio of 3.72, and that “always” influenza vaccinators are 24.7% more likely to also receive COVID-19 vaccination.^13^,^14^

Three respondents cited anecdotal evidence from healthcare workers (HCW) as major influences in their opinions on vaccination, both positively and negatively. Similarly, other work has found that in an ED setting, patients’ willingness to be vaccinated has been associated with the vaccination status and opinions on self-vaccination of the HCWs directly treating patients, with unvaccinated HCWs being less likely to recommend vaccination to patients.^41^–^43^ The HCWs have been shown to share similar reservations about both influenza and COVID-19 vaccines as non-HCWs, including underestimating the impact of influenza infection, underestimating the effectiveness of the vaccine, and overestimating the severity of vaccine side effects, with nurses reporting greater vaccine hesitancy than physicians.^44^–^46^

Notably, most participants in this study are from an underserved population that may face greater challenges regarding access to care. Most of them lacked a primary care clinician, many participants lacked insurance, and 16.3% reported having no steady home or experienced housing insecurity. Additionally, a higher percentage of participants in our study identified as a racial or ethnic minority in comparison to the general public, which is representative of typical ED patient demographics.^24^,^25^ All these factors have been previously associated with reduced influenza vaccine uptake.^16^,^19^,^23^,^34^,^47^ Unlike prior work which highlighted perceived cost and lack of knowledge about where to receive vaccination as significant barriers to access, particularly for uninsured patients, our results reveal that most participants felt they had access to influenza vaccines.^34^,^37^ One reason for this difference could be expanded vaccine access and knowledge of public vaccination sites due to COVID-19.

Directly addressing misinformation related to vaccine hesitancy (such as the idea that the influenza vaccine causes influenza) has historically been difficult.^48^,^49^ Identifying the most effective messages and method of delivery of messages is paramount. Nyhan et al found that directly addressing misinformation related to the pneumococcal and measles, mumps, and rubella vaccines among those with vaccine hesitancy resulted in a greater resistance to vaccination, even if patients recognized their opinions were based on misconceptions.^48^ Conversely, our approach in the highly successful PROCOVAXED trial was to provide direct, neutral, explanatory information on COVID-19 vaccines through short videos, flyers, and scripted messaging to ED patients from physicians who look like them (eg, Black physicians on videos delivered to vaccine-hesitant Black patients). We found that our messaging platforms doubled vaccine acceptance and uptake in unvaccinated ED patients.^31^

Overall, our findings suggest that future educational interventions to address influenza vaccine hesitancy should provide accurate, neutral, and easily understandable information about the vaccine’s safety and effectiveness. The role of clinicians in framing and delivering the information is likely important and requires clinician buy-in. Clinicians should seek to understand specific negative experiences the patient may have had or heard about, with the goal of addressing them in a nonjudgmental, supportive, and clarifying way. Interventions should also include information about how the vaccine protects against illness, how it is produced, and why there is a need for annual inoculation. Patients may benefit from materials that appropriately reflect their own health status, community, and cultural background, and/or relatable anecdotes, as well as examples of how the influenza vaccine can be beneficial to a given individual.

We have developed ED-specific influenza vaccine messaging platforms based on this work and have conducted a cluster randomized controlled trial to determine whether the implementation of these platforms is associated with greater influenza vaccine acceptance and uptake in unvaccinated ED patients.^50^

## LIMITATIONS

This study should be considered in the context of its limitations. Our findings may be limited by sample size. Although there was no predetermined enrollment target for this substudy, we attempted to mitigate this limitation by enrolling participants in the parent study until thematic saturation was achieved. While we did not assess for thematic saturation within the influenza substudy, interviews including influenza questions ranged from 9 to 17 per study site, and recent literature suggests 73% to 92% of codes emerge within the first 6 to 12 interviews.^51^

Additionally, our influenza responses still spanned the same breadth of themes and codes as the COVID-19 responses as well as new emergent codes, suggesting we approached thematic saturation.

An additional limitation may be the timing of data collection, which occurred at the height of the Delta variant of the COVID-19 pandemic, and just prior to the surge of the Omicron variant of COVID-19 in the United States. The relatively uncertain and higher-risk nature of the Delta variant may have influenced attitudes and beliefs regarding vaccination for both COVID-19 and influenza; these beliefs may shift as the expected severity of COVID-19 disease has become more mild.^52,53^ Additionally, the greater emphasis on vaccination for COVID-19, and increased accessibility of vaccination sites may have resulted in an improved perception of access to influenza vaccination in our cohort, in comparison to past years.

As part of a larger study, discussions with each participant occurred within the context of an in-depth interview about the COVID-19 vaccine. Because not all participants were able to complete the dedicated influenza subcomponent of the interview due to time constraints, selection bias may have been introduced in the current analysis, which includes only those who provided influenza-related information. Additionally, all participants were unvaccinated against COVID-19, thus likely representing a subset of the ED patient population that is relatively more vaccine-hesitant. Conversely, because these data were collected at a time when information about the COVID-19 vaccine was not yet widely available, our qualitative understanding of the intersection between influenza and COVID-19 vaccine hesitancy may add additional value to the literature. In addition, participants may have been primed to respond in a particular way (whether positively or negatively) toward questions about influenza illness and the influenza vaccine as they had been prompted to think about and share their fears, concerns, or other negative thoughts and feelings toward the COVID-19 vaccine in the interview. Lastly, while our study was conducted at four sites located in different regions throughout the country, all sites were Level I trauma centers serving primarily urban populations. Therefore, it is possible demographic and selection biases may have contributed to our data and subsequent analyses and may lack generalizability to rural patient populations.

## CONCLUSION

This multicenter, qualitative ED-based study is the first to our knowledge to describe in-depth factors contributing to influenza vaccine hesitancy in adult COVID-19 unvaccinated patients. Despite being knowledgeable about the purpose and mechanism of the vaccine, most patients cited concerns about lack of vaccine effectiveness, fear of side effects, and lack of concern regarding influenza illness. Our findings offer valuable insights that can inform future ED-based vaccine education interventions, particularly for vulnerable patient populations who may not have access to healthcare through other pathways.

## Supplementary Information





## Figures and Tables

**Table 1:** Semistructured interview guide used to interview study subjects In a qualitative study on perspectives about influenza vaccination among COVID-19 unvaccinated patients in the emergency department.

Primary Interview Question	Follow-Up Questions
Have you ever heard of flu vaccines?	If yes: What have you heard?
Have you ever had a flu vaccine?	If yes: When was the last time you got it? Where did you get it? If no: Have you ever been offered a flu vaccine? If yes: What are the reasons that you did not get it when you were offered? If no: Would you want to get a flu vaccine? If yes: What has made it hard for you to get? If no: Why would you not want to get a flu vaccine?
If you were offered a flu vaccine this year, would you accept it?	If yes: Do you know of any barriers to getting a flu vaccine? Have you had a place to get it? If no: What are the reasons you would not accept it? What specific concerns or worries have you had about the vaccine? (eg, side effects)

**Table 2:** Codebook for content analysis used to analyze themes emerging from subject interviews in a qualitative study on perception of influenza vaccination among COVID-19 unvaccinated patients in the emergency department.

Code	Definition/Explanation
Accessing the vaccine	Includes whether they’ve been offered the vaccine, where they were offered it, where they think they could get the vaccine, where they feel most comfortable getting it, and how other people can access it. Also includes any barriers to access.
Experiences	Includes experiences of the patient OR others having the flu and includes experiences of receiving the flu vaccine.
Fears, worries, or concerns	Includes all fears, worries, or concerns regarding the flu vaccine. When coding here, do not double code in other places.
Future intentions	Intent to either accept or refuse the vaccine in the future includes plans to get it later (eg, “I’m going to get it once I’m feeling better”) and accepting the vaccine on the same day as the interview.
Influences (programs and people)	The impact of programs or initiatives related to the vaccine. Includes experiences of others related to the vaccine, may include stories of people deciding to get or not get the vaccine, includes experiences about others getting the vaccine. Also includes being influenced NOT to get the vaccine. Participant needs to explicitly say something along the lines of, “and that is why I am or am not getting the vaccine.” Also include examples of people describing how media is NOT influencing them. Includes: Incentives Information or things that need to happen that would influence vaccine acceptance—this will help inform researchers on best messaging for future communication Mandates/ social pressures Other life circumstances—(or things that get it in the way) things that impact their willingness or ability to get the vaccine
Impact of faith and discussion of faith	Only code if faith religion/faith community is impacting their decision to get vaccinated.
Patient perceptions and understanding of Influenza	Includes instances of individuals referencing their strong immune system. Also includes specific references to people they know who have had the flu. If participant only discusses what they’ve heard but does not discuss the impact of what they have heard, code here.
Patient understanding of the flu vaccine	Includes information participants have heard about the vaccine. If they are describing others’ experiences that they have heard from the news or media, code here and NOT “Experiences”. Includes: Specific references to lack of knowledge—not having heard about the vaccine or don’t know about the vaccine Comparing symptoms of the flu to side effects of the flu vaccine
Sources of information regarding the flu vaccine	Includes references to friends and family, or others that give the participant info. Try not to double code to “Influences.” unless they explicitly also mention how much a particular source influences them above others.

**Table 3:** Demographics of COVID-19 unvaccinated patients in the emergency department who participated in a qualitative study examining perceptions of influenza vaccination.

	*N* (%)
Age in years, median (IQR)	34 (28, 50.5)
Sex	
Male	27 (56.2%)
Female	21 (43.8%)
Hispanic/Latino	
Hispanic/Latino	14 (29.2%)
Not Hispanic/Latino	33 (68.8%)
Not reported	1 (2.1%)
Race	
White	18 (37.5%)
Black	16 (33.3%)
Asian	1 (2.1%)
Mixed Race	3 (6.3%)
Pacific Islander	1 (2.1%)
Native American	1 (2.1%)
Other	5 (10.4%)
Not reported	3 (6.3%)
PCP	
Has PCP	6 (12.5%)
No PCP	34 (70.8%)
Not reported	8 (16.7%)
Housing Status	
Steady home	40 (83.3%)
Housing Insecurity	2 (4.2%)
No steady home	5 (10.4%)
Not reported	1 (2.1%)
Insurance Status	
Medicaid	12 (25.0%)
Medicare	8 (16.7%)
Medicaid and Medicare	1 (2.1%)
Military	1 (2.1%)
Private insurance	13 (27.1%)
ACA Marketplace plan	1 (2.1%)
No insurance	8 (16.7%)
Not reported	4 (8.3%)

**Table 4:** In a qualitative study on perceptions of the influenza vaccine in COVID-19 unvaccinated patients in the emergency department, common themes identified among participant responses to semistructured interviews.

Theme	Subtheme	Example Quote
Past experiences with flu illness and vaccinations	Contracted flu despite vaccination	*I noticed just with my own analysis was like every time I got vaccinated for the flu I ended up getting sick—end up getting the flu. When I didn’t get vaccinated for that I didn’t get the flu. It’s weird. [W008]*
	Experiences with side effects	*I started probably about five years ago. And got sick, real sick after each one. But progressively every time I got it, it was worse. I don’t want the shot anymore. I pretty much stay away from everybody. [W001]*
	Never contracted influenza	*And then like I told you, I’ve never caught the flu. So I mean, why would I get a vaccine for something I never caught. [D001]*
Knowledge	Understanding the flu vaccine	*That I’ve heard they’re injecting you with the virus to help get your body immune to it. [D008]*
	Comparing flu vs. COVID vaccines	*Well, no, well it’s a little different because I’ve been taking it for years. And well it’s a little different because it has been around for more years. [SSPAN001]*
	Sources of information	*And one nurse practitioner, she said that she’ll never get a flu shot again. And I said, well, why won’t you get the flu shot? Because you’re supposed to be a doctor and this kind of stuff. [S005]* *I think I heard that in the news. They said that people with influenza they would get the vaccine and they wouldn’t feel well sometimes. [SSPAN006]*
Fears and concerns	Concerns about potential side effects	*I’m scared of catching—I’m scared of what I don’t know. I don’t know what it’s gonna do to me. I don’t know what kind of reaction I might have. [D009]*
	Concerns about effectiveness	*The flu vaccine’s been out for how many years? Probably. I don’t know. Older than I am, and it’s uneffective. People get the flu after they get it. [D006]*
Access to Uptake		*Interviewer: Do you know of any barriers to getting the flu vaccine, like anything that’s ever prevented you from being able to go and get the flu shot* *Participant: Not at all. I get it offered all the time. [J007]*
Future intentions		Interviewer: if you are offered the flu vaccine this year, would you take it? *Participant: I don’t—why would I need it? It’s just—I’ll trust Mother Nature before I trust—Mother Nature’s a lot more complex and a lot more intelligent. [S006]*
