# Supplementary material for: Perspectives on Influenza Vaccination Among COVID-19 Unvaccinated Patients in US Emergency Departments: A Qualitative Study
Source: West J Emerg Med. 2026 Oct 29;27(6):1631–41. doi: 10.5811/westjem.62236 (PMC13761963; doi:10.5811/westjem.62236)
Supplement: Supplementary file 1 [file wjem-27-1631-s001.pdf]

**S1 File: PROCOVAXED Study Network**

| *Group Name(s): PROCOVAXED Network | | | | | | | |
|---|---|---|---|---|---|---|---|
| ***First Name and Middle Initial(s)** | ***Last Name** | ***Suffix (eg, Jr, III)** | Academic Degrees | Institution | Location (city, state/province, country) | Role or Contribution, eg, chair, principal investigator | Group (if more than 1 Group listed in the byline) and/or Subgroup (eg, Steering Committee) |
| Hemal | Kanzaria | | MD, MSc | University of California, San Francisco | San Francisco, CA, USA | Co-investigators | |
| Robin | Kemball | | MPH | University of California, San Francisco | San Francisco, CA, USA | Research Manager | |
| Virginia | Chan | | BS | University of California, San Francisco | San Francisco, CA, USA | Research Coordinator | |
| Amanda | Guth | | MPH | Thomas Jefferson University | Philadelphia, PA, USA | Research Coordinator | |
| Iyaniwura | Olarewaju | | BA | Thomas Jefferson University | Philadelphia, PA, USA | Research Coordinator | |
| Sophie C. | Morse | | BS | University of Washington | Seattle, WA, USA | Research Coordinator | |
| Ashini | Patel | | BS | Thomas Jefferson University | Philadelphia, PA, USA | Research Coordinator | |
| Kevin | Schaeffer | | BS | Thomas Jefferson University | Philadelphia, PA, USA | Research Coordinator | |
| Dylan | Grau | | BS | Thomas Jefferson University | Philadelphia, PA, USA | Research Coordinator | |
| Abir | Arab | | BS | Thomas Jefferson University | Philadelphia, PA, USA | Research Coordinator | |
| Phillip | Watts | | BA | Thomas Jefferson University | Philadelphia, PA, USA | Research Coordinator | |
| Hailey | Shughart | | BA | Thomas Jefferson University | Philadelphia, PA, USA | Research Coordinator | |
| Erica | Walker | | BS | Duke University | Durham, NC, USA | Research Coordinator | |
| Chris | Klasson | | BA | Duke University | Durham, NC, USA | Research Coordinator | |

Supplemental Online Content: Nonauthor Collaborators
*First name, last name, and suffix (if applicable) are required and will appear in PubMed.

| ***First Name and Middle Initial(s)** | ***Last Name** | ***Suffix (eg, Jr, III)** | Academic Degrees | Institution | Location (city, state/province, country) | Role or Contribution, eg, chair, principal investigator | Group (if more than 1 Group listed in the byline) and/or Subgroup (eg, Steering Committee) |
|---|---|---|---|---|---|---|---|
| Bryan | Yam | | | Duke University | Durham, NC, USA | Research Coordinator | |
| Sophie | Finkelstein | | | Duke University | Durham, NC, USA | Research Coordinator | |
| Harrison | Chen | | | Duke University | Durham, NC, USA | Research Coordinator | |
| Nikki | Daniels | | | Duke University | Durham, NC, USA | Research Coordinator | |
| Joshua | Sarafian | | | Duke University | Durham, NC, USA | Research Coordinator | |
| Lauren | Howard | | | Duke University | Durham, NC, USA | Research Coordinator | |
| Lea | Alali | | BS | Duke University | Durham, NC, USA | Research Coordinator | |
| Glory | Agun | | | Duke University | Durham, NC, USA | Research Coordinator | |
| Emmanuel A. | Chan | | MS, MED | Duke University | Durham, NC, USA | Research Coordinator | |
| Anna | Covington | | BS | Duke University | Durham, NC, USA | Research Coordinator | |
