# Supplementary material for: Perspectives on Influenza Vaccination Among COVID-19 Unvaccinated Patients in US Emergency Departments: A Qualitative Study
Source: West J Emerg Med. 2026 Oct 29;27(6):1631–41. doi: 10.5811/westjem.62236 (PMC13761963; doi:10.5811/westjem.62236)
Supplement: Supplementary file 2 [file wjem-27-1631-s002.docx]

**S2 Table. Research team and reflexivity**

| **Initials** | **Interviewer/**  **Facilitator; Coder** | **Credentials** | **Occupation** | **Gender** | **Experience and Training** | **Relationship with Participants (for interviewers)** |
| --- | --- | --- | --- | --- | --- | --- |
| CL | Coder | BS | Clinical Research Coordinator | Female | Experience with qualitative & quantitative research methods, study coordination, and data management. | N/A |
| JTW | Coder | N/a | Clinical Research Assistant | Male | Experience with qualitative and quantitative research methods. | N/A |
| RG | Interviewer | MPH | Research Scientist | Female | Experience with qualitative and quantitative research methods. | Relationship established at time of consent. Participants did not have any information about the researcher outside of the researcher’s role on the study team. |
| AMG | Interviewer | MPH | Clinical Research Coordinator | Female | Experience with qualitative and quantitative research methods. | Relationship established at time of consent. Participants did not have any information about the researcher outside of the researcher’s role on the study team. |
| CLC | Interviewer for both English and Spanish speakers | BA. Fluent, credentialed Spanish speaker | Clinical Research Coordinator | Female | N/A | Relationship established at time of consent. Participants did not have any information about the researcher outside of the researcher’s role on the study team. |
| AMP | Interviewer | MScGH | Clinical Research Coordinator | Female | Experience with qualitative & quantitative research methods, study coordination, and data management. | Relationship established at time of consent. Participants did not have any information about the researcher outside of the researcher’s role on the study team. |
| ERW | Interviewer | MHA | Clinical Research Coordinator | Female | Experience with qualitative, quantitative research methods, study coordination, and data management. | Relationship established at time of consent. Participants did not have any information about the researcher outside of the researcher’s role on the study team. |
